# New Biparietal Bipolar Catheter Prototype for Hybrid Atrial Fibrillation Ablation

**DOI:** 10.1177/1556984520981025

**Published:** 2021-01-07

**Authors:** Francesco Matteucci, Bart Maesen, Carlo De Asmundis, Gianmarco Parise, Linda Renata Micali, Gabrielle Tuijthof, Peter Gerits, Kevin Vernooy, Jos G. Maessen, Mark La Meir, Sandro Gelsomino

**Affiliations:** 1 118066 Cardiovascular Research Institute Maastricht - CARIM, Maastricht University Medical Center, Maastricht, The Netherlands; 2 5211 Cardiothoracic Department, Maastricht University Hospital, Maastricht, The Netherlands; 3 60201 Cardiothoracic Department, Brussels University Hospital, Brussels, Belgium; 4 5211 IDEE Engineering, Maastricht University, Maastricht, The Netherlands; 5 118066 Maastricht Instruments BV, Maastricht, The Netherlands

**Keywords:** atrial fibrillation, catheter ablation, surgical ablation, hybrid ablation

## Abstract

**Objective:**

To evaluate the size and depth of linear lesions by in vitro testing with a custom-made radio frequency biparietal bipolar ablation catheter in a single-stage setting.

**Methods:**

A custom-made catheter was created to generate linear lesions around the left atrium and pulmonary veins of an ex vivo pig. Two frames were made, 1 epicardial and 1 endocardial. A continuous copper braid electrode and an alignment system consisting of 2 parallel rows of neodymium magnets were embedded in a flexible plastic support. After 24 hours of formalin conservation, samples of the left atrium of a freshly slaughtered pig were sliced in a cryotome, thus obtaining a sequence of 100-µm thick layers extending from the endocardial to the epicardial side. After being digitized through a scanner, these layers were evaluated using morphometric computer software. For each slice, we evaluated the maximum length of the lesions, the maximum epicardial length, the maximum endocardial length, the total area of the lesion, and the total volume.

**Results:**

Forty transmural lesions from 40 specimens were obtained. The results were the following (the number in parenthesis is the interquartile range in mm): lesion maximum length (*L*_MAX_) was 7.297 mm (0.006), epicardial maximum length (*L*_EPI_) was 7.291 mm (0.014), and endocardial maximum length was 7.291 mm (0.018). The total area and total volume were 1018.50 ± 36.51 mm^2^ and 101.85 ± 3.65 mm^3^, respectively.

**Conclusions:**

Our prototype showed very promising results. The next step will be to enhance the design for clinical application.

**Central Message**

We present a new prototype based on biparietal bipolar radiofrequency technology. The in vitro tests showed 100% transmurality. Further experiments are needed to verify whether an actual hybrid biparietal bipolar ablation is feasible in clinical practice.

## Introduction

Atrial fibrillation (AF) is the most common arrhythmia affecting more than 40 million people worldwide.^1-3^ The surgical approach has evolved over the years from the original cut-and-sew Cox maze procedure to the recently introduced hybrid ablation (HA) procedure, which is a combination of a transcatheter cardiac ablation and a video thoracoscopic surgical approach, employed in 1 or multiple stages to overcome the limitations of each procedure.^4^ This simultaneous use of the 2 procedures serves to combine their advantages in improving the transmurality of lesions.

Bipolar clamps and pens are the tools usually used with this technique to isolate the left atrium posterior wall, which is the area most commonly involved in generating ectopic electric impulses.^5^ Nonetheless, despite promising early results with HA, freedom from AF without antiarrhythmic drugs is still suboptimal, especially in patients with long-standing persistent AF^6,7^ due to the occurrence of important electrical and structural remodeling.^4,8^ Thus, there is a unanimous consensus of the need for new optimized tools to improve the success rate of AF ablation thus leading to long-term stable sinus rhythm.^9^

The present work aims to describe and test a new biparietal bipolar catheter prototype in vitro for hybrid AF ablation.

## Methods

### Prototype

**Fig. 1:**
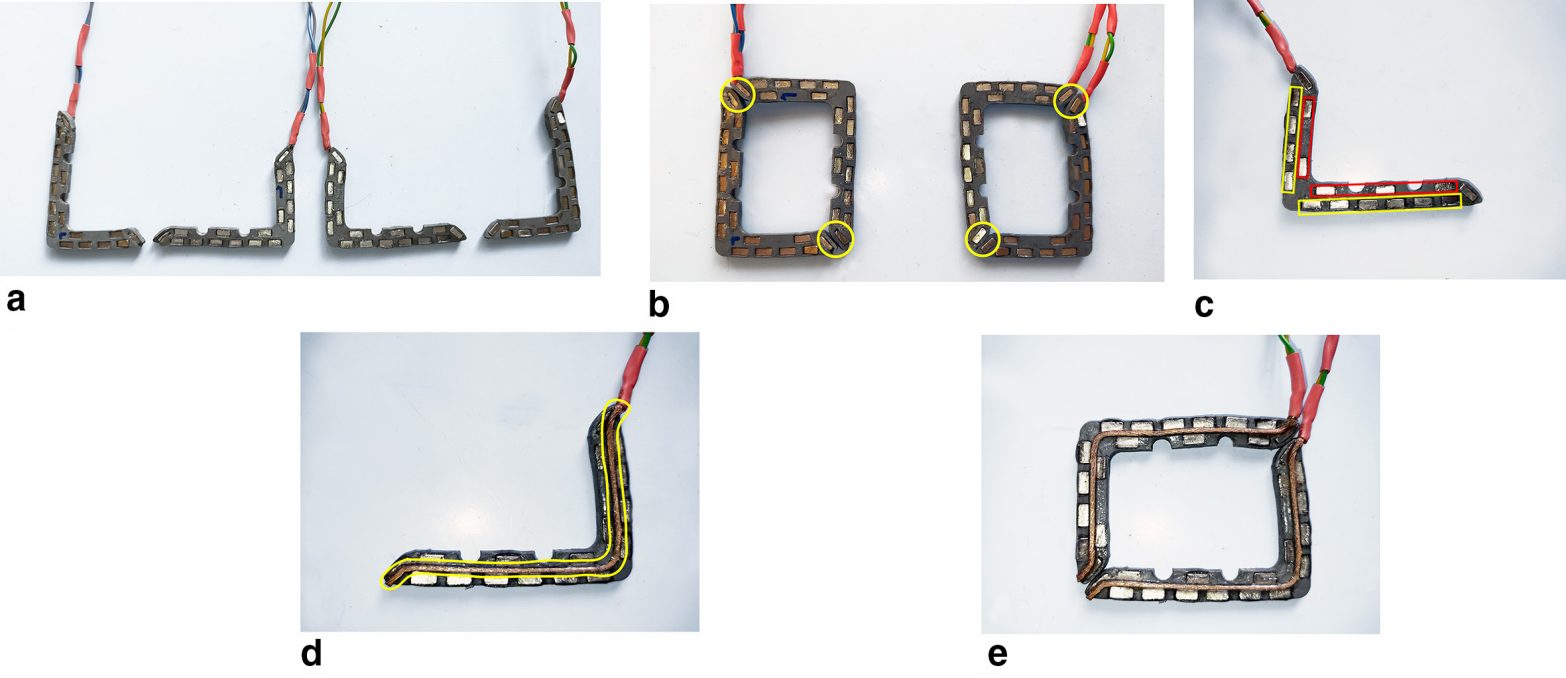
(**a**) External view of the prototype consisting of 4 frames. (**b**) Frames combined 2 by 2 by magnetic lock. (**c**) Magnet placement along with the frame: the yellow boxes show the outside row, and the red one shows the inner row. (**d**) A detail of the internal view of the single frame. The yellow path highlights the copper braid electrode and its placement. (**e**) Complete view of the internal part of the frame.

The new prototype (patent application no. 18206993.0) was created in collaboration with Instrument Development Engineering & Evaluation (IDEE, Maastricht University, NL). The prototype includes 4 identical plastic, flexible L-shape frames (L 50 mm, W 35 mm, H 3 mm; Fig. 1a). These frames are connected 2 by 2 by a couple of neodymium iron boron (NdFeB) magnets (Supermagnete, Webcraft GmbH, Gottmadingen, Germany) embedded in the margin of the opposite shape (Fig. 1b). The resulting couples of frames are placed side by side, interposing the tissue between them.

The frames are shaped by a laser precision machine (Speedy 300 Flexx, Trotec Laser, Marchtrenk, Austria) to guarantee a specular match between them. Furthermore, 9 magnetic elements, arranged in 2 parallel rows (6 external and 3 internal) over the long branch and 6 for the shorter one (4 external and 2 internal), are included in the frame (Fig. 1c). For the best alignment and spatiality of these magnetic elements, we made a shallow laser incision in the surface and, subsequently, we made full-thickness incisions to allow for the placement of the magnetic elements.

The dimensions of the neodymium iron boron (NdFeB) magnet is L 5 × W 2.5 × H 1.5 mm, generating approximately 350 g of magnetization strength from a 1.5 mm axis direction.

On the sides facing the tissue, between the previously mentioned parallel magnet rows, a continuous path was created through a precision laser incision over the single L-shape half-frame. A copper braid with a diameter of 1.2 mm diameter and 8 cm long (Wires.co.uk, Great Dunmow, Essex, UK) was laid down along the entire linear incision that was made through the laser (Fig. 1d–e).

All elements were fixed with cyanoacrylate-based glue. The copper braids were electrically connected with a standard radiofrequency (RF) generator for cardiac ablation.

### Simulator

**Fig. 2:**
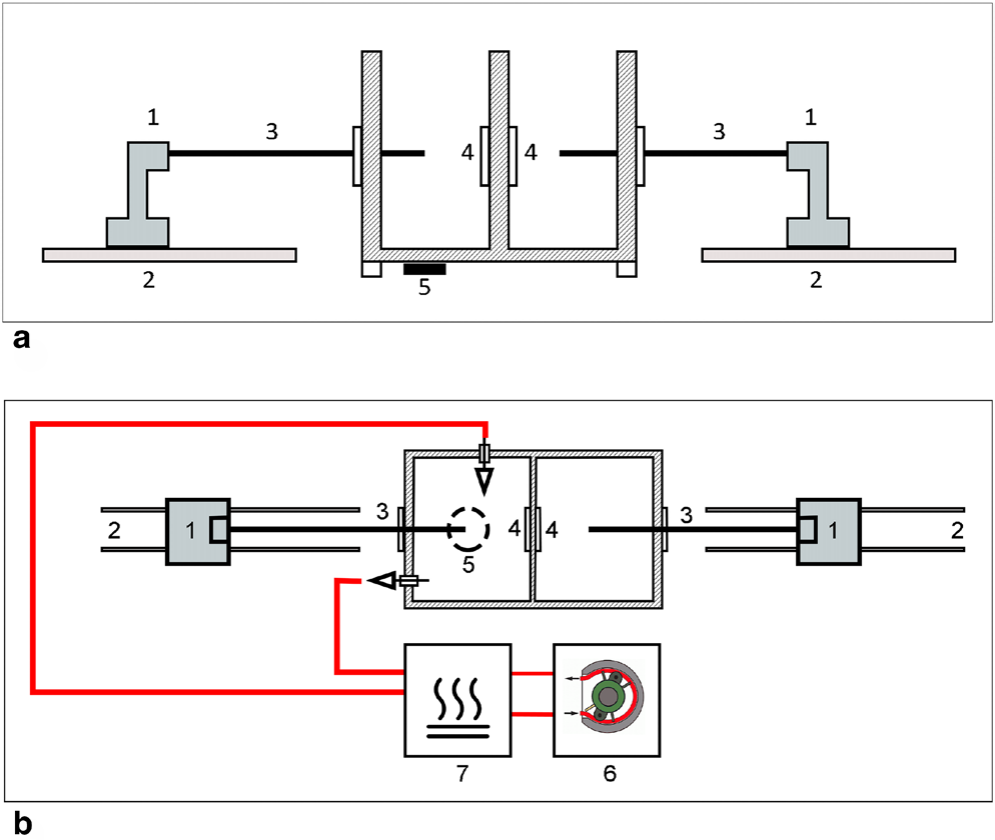
Scheme of ABLA-BOX. (**a**) Lateral view and (**b**) top view. (1) Catheter holder with pressure sensors. (2) Rails where the holders move over. (3) Catheters. (4) Inner tissue holder. (5) Magnetic stirrer motor. (6) External pump. (7) External heating exchange device.

For the testing of the prototype, we used a previously described simulator (ABLA-BOX).^10^ Briefly, it consists of a double chamber plexiglass box mimicking the left atrium with the separation between the 2 chambers representing the left atrial wall. Two rails are placed side by side externally to the box, and a catheter holder with a pressure sensor (AE Sensors, Dordrecht, The Netherlands) is mounted over both rails (Fig. 2a). The chamber mimicking the interior of the atrium comprises an inlet and outlet socket where an external pump (COBE precision blood pump, COBE Cardiovascular Inc., Arvada, CO, USA) is connected to simulate the blood flow. The blood was heparinized (Heparin Leo 5.000 UI/mL. LEO Pharma A/S, Ballerup, Denmark) to reach an ACT time >400 seconds. A heating plate is placed beneath the box to keep the temperature at a physiological level (Fig. 2b). In addition, an external heating exchange device (Bio Cal 370, Medtronic, Minneapolis, MN, USA) is used to keep the circulating blood at a constant temperature. Also, a magnetic stirrer motor (Cyclone 1Y100, HMCEurope, Tuessling, Germany) is placed beneath the plastic chamber, and metallic elements with different shapes are placed into the chambers and driven by the stirrer motor to simulate the turbulence of blood flow typical of an AF condition.

### Samples

**Fig. 3:**
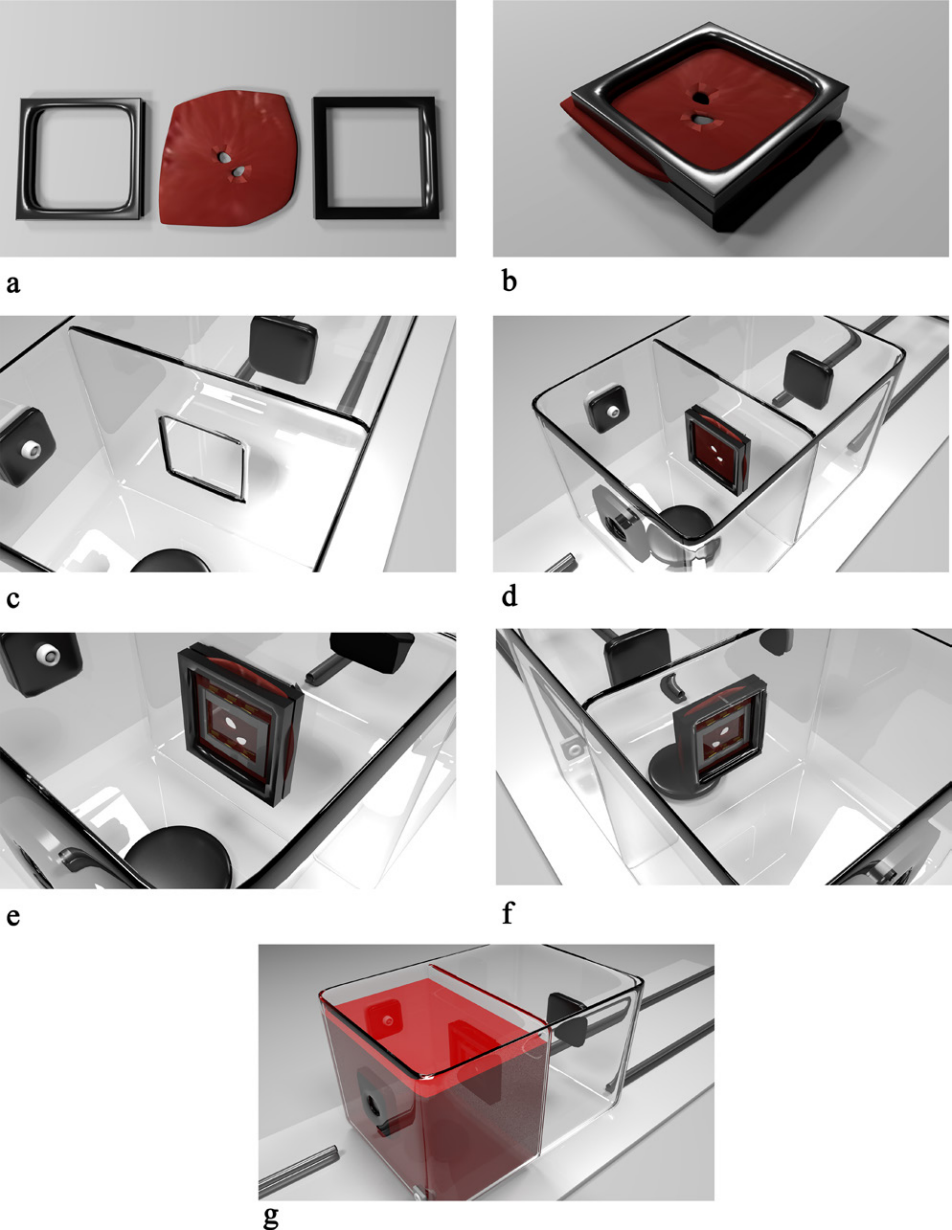
Sequential steps in the placement of the device into the simulator. (**a**) On the left and right side, two 3-dimensional printed frames are depicted. In the middle, the sample is shown. (**b**) The sample is placed between the frames to keep it unfolded. (**c**) A central aperture letting the samples being lapped by the blood (endocardial part), and air (epicardial part) is present on the wall separating the 2 chambers. (**d**) The frames and the sample between them are magnetically fixed to the simulator. (**e–f**) The prototype frames attract each other, squeezing the tissue. (**g**) The chamber mimicking the endocardial part is filled with circulating blood, and the radiofrequency application can start.

**Fig. 4:**
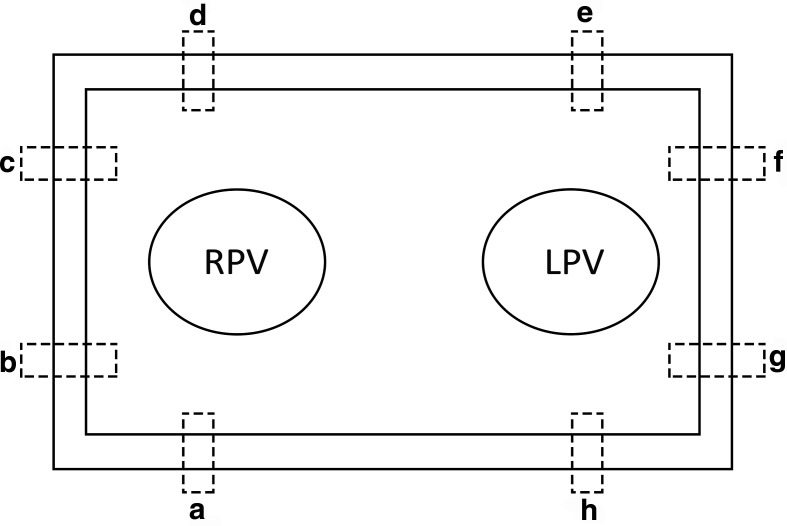
Ablated frame scheme. The prototype surrounded the area of the pulmonary veins (RPV and LPV). From each leg of the ablated linear lesion frame, 2 full-thickness samples of 5 mm in width and 20 mm in length were excised for examination. In total, for every ablated frame, we obtained 8 samples (**a–h**). LPV, left pulmonary vein; RPV, right pulmonary vein.

Fresh porcine hearts with the entire lung/bronchus/trachea were collected. In the laboratory, the left atrium was meticulously isolated maintaining the anatomy of the 2 pulmonary veins of the porcine hearts. The secured anatomical parts were immersed in a transport medium, Roswell Park Memorial Institute 1640 (Sigma-A Samples Aldrich, St. Louis, MO, USA). Then, the tissue was unfolded, and the prototype mounted on both sides (Fig. 3). In total, we used 5 hearts, on which we made 1 ablation each. We studied 40 samples and sliced them into approximately 2000 layers. Two samples of 5 mm width and 20 mm length were excised from each edge of the lesion frame (4) and were analyzed (Fig. 4).

### Experimental Setup

Our console-setup measured tissue conductance and impedance throughout the ablation cycle (50 times per second) and controlled the application of energy to the tissue. The output of the power generator was fixed in bipolar mode to 28.5 W at 114 Ω. The maximum delivery of energy was reached along a constant power zone. The system was set up at 30 to 70 Ω for the tissue impedance or 15 to 30 millisiemens for conductance. The RF delivery automatically stopped when a stable low level of conductance was reached, indicating that a lesion had been created.

The hemodynamic parameters were fixed to 5 L/min for blood flow to mimic standard human cardiac output,^11^ magnetic stirrer to 400 rpm, and heating system to 38 °C.

### Morphometric Evaluation of Myocardial Tissue Ablation

The specimens were put in 10% neutral buffered formalin for 48 hours. The samples were then embedded in the Tissue-Tek OCT, and slices with a thickness of 100 µm were cut using a Leica CM 3050 cryostat (Leica Biosystems, Wetzlar, Germany).

**Fig. 5:**
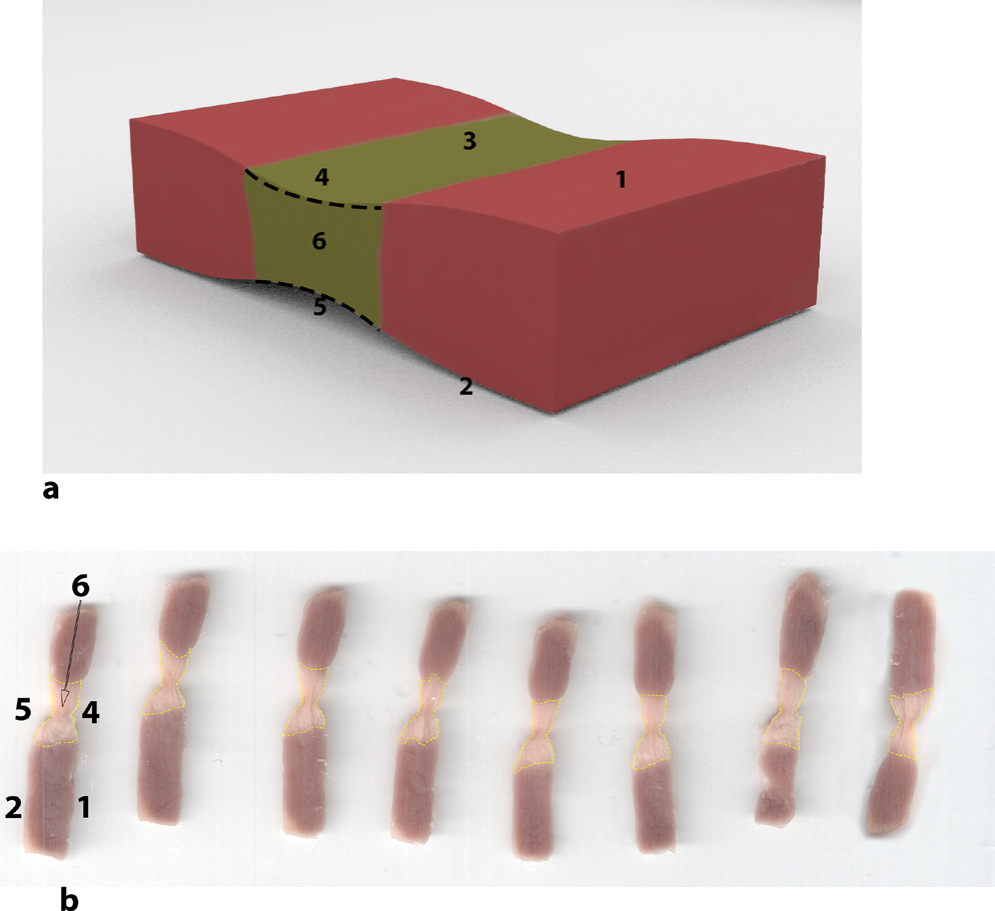
Scheme of excised samples. (**a**) 3-dimensional sketch of the sample; (**b**) sequence sample slices. (1) Epicardial side, (2) endocardial side, (3) linear lesion, (4) maximum epicardial lesion length, (5) maximum endocardial lesion length, and (6) area lesion.

The slices were obtained by cutting the samples perpendicularly to the direction of the lesion so as to assess the existence of possible gaps along with the linear injury. The specimens were laid down on a transparent acetate sheet, dried for 1 hour at room temperature, and finally covered with another transparent sheet to protect them. The sheets were subsequently digitalized through a flatbed scanner (Perfection V39 Scanner, Epson America, Inc., Long Beach, CA, USA) at 600 dpi of resolution. Finally, a Java-based image processing program (Image J version 1.48 software; National Institutes of Health, Bethesda, MD, USA) was used to measure the extension of the lesions. The total area (*A*_tot_) for each sample was calculated by the sum of the area of the single layer. The total volume (*V*_tot_) was quantified by multiplying *A*_tot_ by the fixed slice thickness. The overall lesion length (*L*_max_), including the epicardial (*L*_epi_) and endocardial (*L*_endo_) lengths, were calculated (Fig. 5).

### Statistical Analysis

The normal distribution of data was tested using the Shapiro-Wilk normality test. Means and SDs were calculated for normally distributed variables and median and interquartile range for non-normally distributed variables.

Five randomly selected samples were used to test the intraobserver and interobserver variability between two readers (FM and GP). The κ-statistic was used to evaluate the degree of intraobserver agreement. The tested variables were *A*_tot_ and *L*_max_. A κ of 1 indicates perfect agreement, whereas a κ of 0 indicates chance agreement. All statistical analyses were conducted using SPSS Statistics, version 18.0 (SPSS Inc., Chicago, IL, USA).

## Results

Both the intraobserver and interobserver variability for *A*_tot_ morphometric evaluation were low (*k* = 0.98 and *k* = 0.91, respectively). Likewise, the variability for *D*_max_ evaluation was low (*k* = 0.96 and *k* = 0.92). The thickness of the sample was 4.18 mm ± 0.55. In all specimens, we did not observe discontinuity in lesions across the entire thickness of the tissue (100% transmurality). The overall maximum length of the lesions (*L*_max_) resulted in 7.297 (0.0066) mm. The lengths of the lesions of epicardium (*D*_epi_) and endocardium (*D*_endo_) were equivalent, that is, (7.2916 [0.014] mm and 7.2911 [0.018] mm). The lesion had an area (*A*_tot_) of 1018.50 ± 36.51 mm^2^ and a volume of 101.85 ± 3.65 mm^3^ (Table 1).

**Table 1:** Ablation Lesion Results.

Measurements	
Transmurality (%)	100
*L*_MAX_ (mm)	7.2970 (0.0066)
*L*_EPI_ (mm)	7.2916 (0.014)
*L*_ENDO_ (mm)	7.2911 (0.018)
*A*_TOT_ (mm^2^)	1018.50 ± 36.51
*V*_TOT_ (mm^3^)	101.85 ± 3.65

Abbreviations: *A*_tot_, total area; *L*_endo_, endocardial lesion maximum length; *L*_epi_, epicardial lesion maximum length; *L*_max_, lesion maximum length; *V*_tot_, total volume.

*Note*: Data are expressed as mean ± SD, median (interquartile range), or number (percentage) as appropriate.

## Discussion

The urgent need for new tools for treating AF has been focused on medical companies to develop more efficient and safer systems. Three companies produce most clamps used in the clinic: AtriCure, Medtronic, and Estech. The first one produces the Isolator Synergy Clamp in different configurations of many curvatures. It provides 2 electrodes (7 cm long) embedded in each jaw, and a continuous impedance monitoring system as a marker for the assessment of transmurality. Medtronic is presently on the market with the Cardioblate series. They are all irrigated with saline solution to improve the generation of lesions. They have flexible jaws and an articulating head. The length of the electrodes is 7 cm. Different models are available which may even be inserted through port-sized incisions (BP2 Gemini). Estech owns the COBRA Revolution Bipolar Clamp, characterized by serpentine electrodes embedded in the jaws and real-time temperature measurements. Our prototype differs from current devices because it exploits the magnetic force rather than the closure of the jaws to couple the electrodes. Moreover, its design allows us to make the “BOX” around the PV in a single shot.

The design was based on the following observations:

1. The use of bipolar RF is associated with higher success rates and longer-lasting transmural lesions than monopolar RF.^12-17^ Bipolar technology is capable of creating discrete and transmural lesions without significant contractions or scar development.^18^ Therefore, bipolar radiofrequency is considered the gold standard for AF ablation,^16,19^ either in open-heart or closed-chest procedures.^18,20^ From experience with the MAZE surgical procedure, it has been learned that the highest ablation efficacy is mainly obtained when 1 jaw of the bipolar clamp device is introduced into the atrium with the atrial wall between the jaws of the clamp (epi-endo and endo-epi).^18^ We assumed that the technique could be enhanced by the interposition of tissue between the electrodes and thus allowing all the delivered energy to flow through the tissue, ensuring optimal results in transmurality achievement observation.^21^ Nonetheless, all of the AF ablation systems currently used in the clinic do not provide the interposition of the endo-epicardial surface between the opposite electrodes since they come into contact only with 1 surface (epicardial or endocardial). The closest design to a biparietal ablation is the Estech COBRA fusion (AtriCure Inc., Mason, OH, USA). Nevertheless, it excludes the circulating blood from the ablation surface, folding, and surrounding the atrial tissue into the catheter. However, the RF poles are only in contact with the epicardial surface; thus, the ablation is not biparietal. Also, the bipolar clamp employed epicardially for PV ablation is not actually biparietal since only the PV epicardium is in contact with the jaws.
  Our biparietal concept exploits an endo-epicardial interposition of the tissue between the electrodes, allowing RF to flow throughout the thickness of the tissue. The atrial tissue, interposed like in a “sandwich” between poles, optimizes the electrode-tissue contact to reduce energy dissipation into the blood.^22^ This design leads to a faster and more focal heating between the electrodes.^23^ The focused energy has the advantage that the therapeutic temperature (producing irreversible lesions) is reached quicker and, therefore, the potential risk of injuring the neighboring tissue is lower.^24^
  The RF current density is inversely proportional to the square of the distance between the electrodes,^18,25^ and, consequently the design of the prototype allows for deeper lesions due to the closeness of the electrodes along with its biparietal bidirectional design.
2. In most cases, pulmonary vein isolation and electrogram-based ablation without linear lesions are effective for terminating persistent AF. However, the adding of linear ablations prevents macro re-entrant atrial tachycardia during the overall follow-up.^26^ As a consequence, it is believed that linear lesions can prevent AF recurrence. The endpoint of linear lesions, following the Heart Rhythm Society/European Heart Rhythm Association/European Cardiac Arrhythmia Society consensus statement, is a bidirectional conduction block to avoid macro re-entrant atrial tachycardia recurrence.^27^ Linear lesions with the RF pen can be obtained with the painting technique, consisting of moving the device in an oscillating manner maintaining continuous contact between the electrodes and the tissue, or through the stamping ablation technique, consisting of applying constant pressure to the tissue without movements. Nonetheless, both techniques do not allow to create continuous transmural lesions. With the stamping method, which is more often employed in clinics, our experience has taught us that multiple repeated touches are necessary, and higher pressures are needed. However, the result will be punctual ablation lesions, not continuous ablation lesions, with high odds of leaving undamaged tissue within the lesion line resulting in the ectopic firing.
3. The current pens also are not biparietal because both poles are placed at the catheter tip and are not in contact with the epicardial and endocardial tissue simultaneously. Furthermore, there is only a small catheter tip-to-tissue contact, meaning that only a fraction of total power is effectively delivered to the tissue. These observations may explain why incomplete nontransmural linear lesions are often created with incomplete blocks and gaps that are highly proarrhythmic.^4,28^ Our prototype can perform bipolar biparietal linear lesion in a single stage, and it allows us to make transmural lesions all along with the linear ablation. Therefore, it seems very promising to make the roof and the inferior line in 1 step with PV isolation and to obtain complete box isolation in one step.
4. Although the hybrid ablation procedure is performed together by a cardiologist and a cardiac surgeon, the epicardial step is completed first because it allows for the long-lasting isolation of the pulmonary veins and the creation of additional lines in the left atrium.^29^ Nonetheless, the time-frame between the endocardial and the epicardial steps can vary from a few hours up to 6 months,^30-36^ and some groups perform either one-stage or staged procedures.^37-39^ In our previous experience,^40^ we failed to find any difference in lesion depth between single-stage or endocardial procedures performed after a 60-minute delay or after a 240-minute delay. Notably, overall, no transmural lesion was obtained. In contrast, the in vitro testing of the biparietal bipolar prototype, employing the same ABLA-BOX setup, resulted in 100% transmurality without discontinuity along with the linear ablation. The combination of these findings might suggest an enhanced transmurality related to the biparietal bidirectional design since, in our previous work, we employed only monodirectional catheters.

### Limitations

Our study presents some limitations that need to be pointed out.

1. We have not tested the COBRA Fusion compared with our prototype because it was beyond the aims of the article. This test will be, however, the object of upcoming research.
2. We did not assess the influence of epicardial fat on the effectiveness of the lesions.
  At this point of our research, we are testing the ability of the device to make continuous and transmural linear lesions. We are aware that the amount of epicardial fat may influence the effectiveness of the lesions and are currently studying a method to objectively quantify the amount the fat in the samples so as to test the effect of the amount of fat on the ablation results.
3. We did not study the occurrence of clot formation possibly caused during ablation by the anticoagulation of the circulating blood.

## Conclusions

Our prototype results are very encouraging. The next step will be a design for clinical application. An enhanced biparietal bipolar prototype device showed very promising results, is under study. It seems to be very promising in optimizing the sizing, design, maneuverability, biocompatibility, and implantability. We are currently also testing the implementation of electromagnetism which would allow us to enhance targeted ablation by providing a selective magnetic coupling only in areas of interest.

Further experiments will be needed to verify whether an actual hybrid biparietal bipolar ablation is feasible in clinical practice.

## Supplemental Material

Presentation S1 - Supplemental material for New Biparietal Bipolar Catheter Prototype for Hybrid Atrial Fibrillation Ablation Click here for additional data file. Supplemental material, Presentation S1, for New Biparietal Bipolar Catheter Prototype for Hybrid Atrial Fibrillation Ablation by Francesco Matteucci, Bart Maesen, Carlo De Asmundis, Gianmarco Parise, Linda Renata Micali, Gabrielle Tuijthof, Peter Gerits, Kevin Vernooy, Jos G. Maessen, Mark La Meir, and Sandro Gelsomino in Innovations: Technology and Techniques in Cardiothoracic and Vascular Surgery
