# Supplementary material for: New Biparietal Bipolar Catheter Prototype for Hybrid Atrial Fibrillation Ablation
Source: Innovations (Phila). 2021 Jan 7;16(2):181–7. doi: 10.1177/1556984520981025 (PMC8108111; doi:10.1177/1556984520981025)
Supplement: Presentation S1 - Supplemental material for New Biparietal Bipolar Catheter Prototype for Hybrid Atrial Fibrillation Ablation [file sj-pptx-1-inv-10.1177_1556984520981025.pptx]

## Slide 1
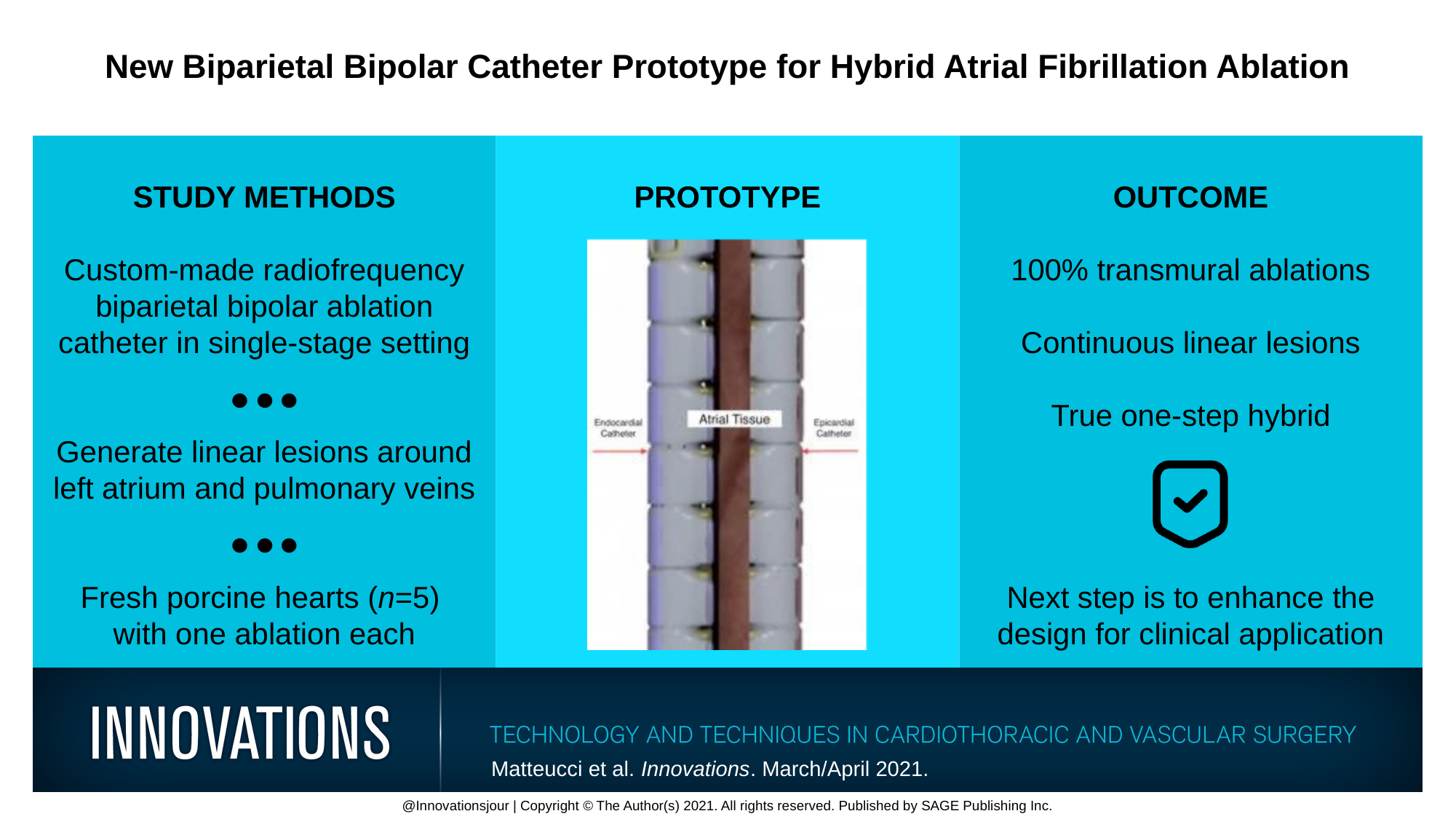

# New Biparietal Bipolar Catheter Prototype for Hybrid Atrial Fibrillation Ablation
STUDY METHODS
Custom-made radiofrequency biparietal bipolar ablation catheter in single-stage setting
Generate linear lesions around left atrium and pulmonary veins
Fresh porcine hearts (n=5)
with one ablation each
PROTOTYPE
OUTCOME
100% transmural ablations
Continuous linear lesions
True one-step hybrid
Next step is to enhance the
design for clinical application
Matteucci et al. Innovations. March/April 2021.
@Innovationsjour | Copyright © The Author(s) 2021. All rights reserved. Published by SAGE Publishing Inc.
